# *POLG* Gene Variants in Cervical Cancer Patients and Their Associations with Clinical and Pathomorphological Tumor Characteristics

**DOI:** 10.3390/jcm10091838

**Published:** 2021-04-23

**Authors:** Ieva Golubickaite, Rasa Ugenskiene, Egle Ziliene, Jurgita Beniusyte, Arturas Inciura, Lina Poskiene, Elona Juozaityte

**Affiliations:** 1 Department of Genetics and Molecular Medicine, Lithuanian University of Health Sciences, 44307 Kaunas, Lithuania; rasa.ugenskiene@lsmuni.lt; 2 Institute of Oncology, Lithuanian University of Health Sciences, 44307 Kaunas, Lithuania; egle.ziliene@lsmuni.lt (E.Z.); jurgita.beniusyte@stud.lsmu.lt (J.B.); arturas.inciura@lsmuni.lt (A.I.); elona.juozaityte@lsmuni.lt (E.J.); 3 Department of Pathological Anatomy, Lithuanian University of Health Sciences, 44307 Kaunas, Lithuania; lina.poskiene@lsmuni.lt

**Keywords:** *POLG*, SNV, cervical cancer, tumor phenotype, outcome

## Abstract

Cervical cancer is one of the most common cancers in women worldwide. Human papillomaviruses are known to be the main, but not the only risk factor, of this cancer type. Despite all the knowledge on this cancer type, it is still a challenge to predict the course of the disease, and therefore, minimally invasive biomarkers are needed. This study aimed to analyze single-nucleotide variants in the *POLG* gene and assess the associations with tumor phenotype and patient outcome. A total of 172 cervical cancer patients were included in this study. Clinical and tumor data were gathered from medical records retrospectively. Single nucleotide variations were determined using TaqMan probes with Real-Time PCR. Significant associations between *POLG* rs3087374 and cervical cancer patients’ tumor histological type, stage, and tumor size were determined. The CA genotype and A allele of rs3087374 increased the probability of adenocarcinoma histological tumor type, IIIA stage, and T3 tumor size compared to CC genotype and C allele, respectively. Furthermore, patients with AA genotype in rs2072267 had longer metastasis-free survival than those with the GG genotype. Our data suggest that mitochondrial polymerase gamma encoded by nuclear *POLG* gene is important for specific tumor phenotype formation and patient outcome in cervical cancer.

## 1. Introduction

Cervical cancer ranks as the fourth most common cancer in women, with almost 0.6 million cases worldwide reported in 2018. Moreover, with 0.3 million cases, it was the fourth leading cancer death cause among women in 2018. This cancer is commonly diagnosed for middle-aged women and is more common in less developed countries resulting in 84% of all cases and 88% of all cervical cancer deaths in lower-resource countries [1]. The human papillomavirus (HPV) infection is known to play an important role in this type of cancer as it is the main cause and a trigger in cervical carcinogenesis [2]. Therefore the HPV vaccination is used to prevent and reduce the risk of cervical cancer [3]. It was shown that countries that have achieved high vaccination coverage observed declines in HPV prevalence and high-grade lesions [4]. For example, Australia was the first one to report HPV vaccination success with a decrease in high-grade cervical abnormalities after its population-wide HPV vaccination implementation [5]. Additionally, HPV screening is also performed and has been shown to be more effective than cytology testing, allowing extension of screening intervals to at least 5 years [6]. Early detection is crucial as the disease can be managed and treated before becoming advanced [7]. In the US, preventive screening programs showed great success as the cervical cancer incidence and mortality decreased significantly, and the majority of cervical cancer-related deaths occurred among females who had not been appropriately screened or treated. Therefore cervical cancer now ranks as the 18th most common cause of cancer in the US [8]. However, not all females are screened or not screened in time, and this leads it to be the most common cause of cancer-related deaths among women in more than 40 countries. Most of them are considered to be low-income or lower-middle-income countries [9]. Although screening for cervical cancer is now widely available, there is still a need for studies on new, highly available biomarkers that would help predict the disease outcome in early stages and in the least invasive and more accessible way.

Single nucleotide variants have been investigated as promising biomarkers for various diseases and conditions. In the majority of published studies, nuclear genes, coding for tumor suppressors and proto-oncogenes, were under investigation; conversely, nuclear genes, responsible for mitochondrial proteins, were rarely under consideration. However, mitochondria are also important for cancerous processes as it is cell powerhouse. Furthermore, it is known that cancerous cells require more energy due to glycolytic energy production, also known as the Warburg effect [10]. Nuclear genes encode the majority of proteins required for mitochondrial function. For example, the only functioning DNA polymerase in mitochondria important for DNA replication and repair is polymerase gamma encoded by the nuclear *POLG* gene [11]. We hypothesize that SNVs in *POLG* might be important in cervical carcinogenesis. To our best knowledge, no previous investigations on *POLG* SNV in cervical cancer patients have been reported so far. In this study, we aimed to analyze four *POLG* SNVs and to evaluate their associations with clinical and pathomorphological data.

## 2. Materials and Methods

### 2.1. Patients and Samples

In total, 172 female patients with cervical cancer as a primary disease were included in this study. The inclusion criteria were: cervical cancer diagnosis as a primary disease, age (older than 18 years of age), signed consent form, availability of clinical and tumor data. Patients that did not consent to participate in the study; had other primary diagnosis or unavailable clinical or tumor data were excluded from the study. The research was carried out at the Institute of Oncology, Lithuanian University of Health Sciences (LUHS) from 2015 to 2021. This study was approved by the Kaunas Regional Biomedical Research Ethics Committee (No. BE-2-10 and P1-BE-2-10/2014). After patients were informed about the study and signed the consent form, their blood samples were collected. Clinical and tumor data were gathered from medical records retrospectively. The date of cervical cancer diagnosis was considered as a time zero. The latest censoring date was on 1 November 2020.

### 2.2. DNA Extraction and Genotyping

The *POLG* gene was selected for this study because of its importance for mitochondria as it encodes the alpha subunit of polymerase gamma (pol γ). Polymerase gamma functions in the mitochondria and it is the only polymerase that is involved in mtDNA replication and repair [11]. *POLG* gene mutations were previously detected in breast and colorectal cancer studies [12,13,14]. For that reason, we were interested in these polymorphisms in cervical cancer and chose to analyze 4 SNPs in the *POLG* gene (rs3087374, rs2307441, rs2072267, rs976072) that have not been widely analyzed before. We selected two SNPs (rs3087374 and rs2307441) that were rare (frequency 0.075 and 0.039, respectively), and two (rs2072267 and rs976072) that were located in non-coding DNA regions (frequency 0.465 and 0.373, respectively) common in the general population [15]. DNA extraction and genotyping took place at the Institute of Oncology, LUHS. DNA was purified using GeneJet Genomic DNA purification kit (Thermo Fisher Scientific, Vilnius, Lithuania, Cat. K0721). Single nucleotide variants in the *POLG* gene were determined using TaqMan^®^ probe SNP Genotyping Assay (Thermo Fisher Scientific, Bleiswijk, Netherlands, Cat. #4351379). The genotyping was carried out using a QuantStudio 3 Real-Time PCR System (Thermo Fisher Scientific, Waltham, MA, USA, Cat. #A28137). Reactions were assembled into a total volume of 12 μL, which included: 15 ng of DNA, 6.125 μL of TaqMan Universal Master Mix (Thermo Fisher Scientific, Bleiswijk, Netherlands Cat. #4304437), 0.625 µL of TaqMan SNP Genotyping Assay, and nuclease-free water. No template control (nuclease-free water) was used to ensure that the reaction was performed without contamination. The standard genotyping PCR program was used. Genotypes were determined according to VIC and FAM fluorescence intensity.

### 2.3. Statistical Analysis

Statistical analysis was performed with IBM SPSS Statistics 22 (SPSS Inc., Chicago, IL, USA) software package. Associations between genotyping data and clinical or tumor features were assessed with Pearson’s Chi-square or Fisher’s Exact tests and binary logistic regression. The Kaplan–Meier and Cox regression analysis was applied to estimate survival; the curves were generated using Lifelines [16]. The differences between survival curves were determined with a log-rank test. The level of significance was set to *p*  <  0.05.

## 3. Results

### 3.1. Patient and Tumor Characteristics

**Figure 1:**
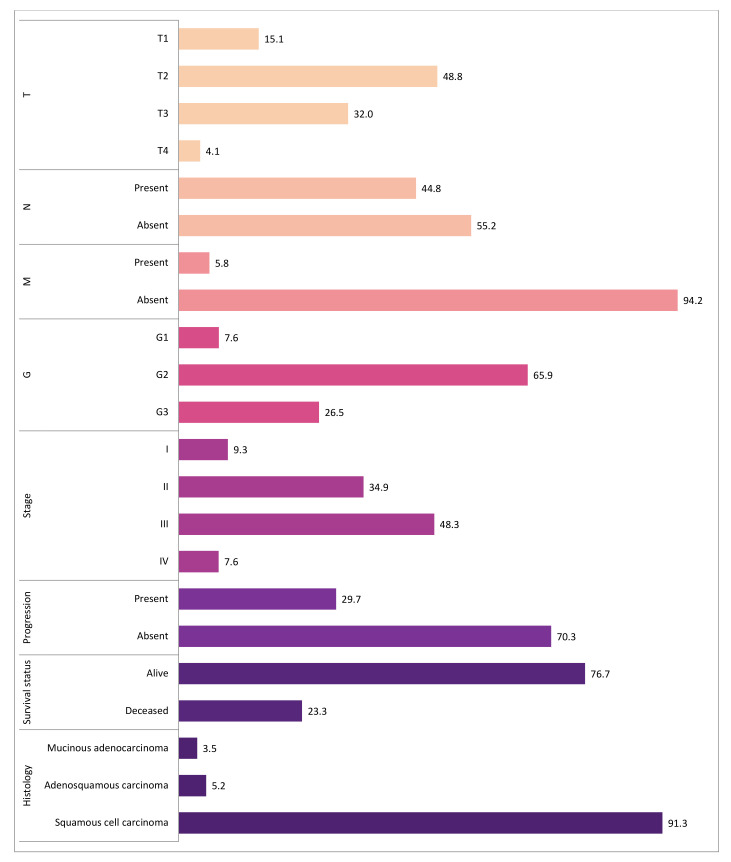
The distribution of tumor and disease characteristics. The bars represent the percentage distribution of tumor and clinical characteristics such as T, pathological tumor size; N, cancerous nearby lymph nodes; M, distant metastasis; G, tumor differentiation grade; stage; progression; survival status; histological tumor type. There were missing data (*n* = 2) in the tumor differentiation grade (G) category. The different color indicates different characteristic.

Our study included 172 female patients with cervical cancer. The age at the time of diagnosis ranged from 22 to 83 years (median 56 years). Most tumors presented with G2 differentiation grade (65.9%) and T2 tumor size (48.8%). The majority of patients had negative lymph nodes (55.2%) and had no distant metastasis (94.2%). There were missing data (*n* = 2) in the tumor differentiation grade category, however, we believe this did not have any significant effect on the results. The detailed information on tumor and disease characteristics is presented in Figure 1 and Supplementary Materials Table S1.

### 3.2. Genotype and Allele Frequencies

The total count and frequencies of *POLG* genotypes and alleles are presented in Table 1. All studied genotypes were in Hardy–Weinberg equilibrium.

### 3.3. Association Analysis

We analyzed possible associations between *POLG* polymorphisms in genotype and allele models and available clinical and tumor data with Pearson’s Chi-square test. However, only one SNP, rs3087374, presented statistically significant results. rs3087374 was associated with adenocarcinoma histological tumor type (*p* = 0.019), T3 tumor size (*p* = 0.025), and stage IIIA (*p* = 0.000) (Table 2). All non-significant associations are presented in the Supplementary Material Table S2–S4. In the allelic model, only rs3087374 A allele was associated with adenocarcinoma histological tumor type (*p* = 0.040), T3 tumor size (*p* = 0.044), and stage IIIA (*p* = 0.001). No other significant associations were determined.

Furthermore, the logistic regression analysis was done to determine possible associations between single nucleotide variations, patient clinical data, and tumor pathomorphological parameters. In univariate analysis, following the adjustment for age at the diagnosis, significant associations were established. That was followed by multivariate logistic regression analysis in the additional model No. 2, including additional confounding factors such as T3 tumor size, IIIA stage, or adenocarcinoma histological tumor type (for more details, please see Table 3).

After logistic regression analysis, it was found that patients with the POLG rs3087374 CA genotype had a 4.5 times higher probability for adenocarcinoma histological tumor type than patients with CC genotype (*p* = 0.034; 95% CI 1.120–18.012). This association remained significant in a multivariate analysis Model No. 2 Table 3 (OR = 4.564 *p* = 0.043; 95% CI 1.050–19.842) (Table 3). Furthermore, the carriers of the A allele also had a 4.5 times higher probability for adenocarcinoma than non-carriers (*p* = 0.034; 95% CI 1.120–18.012). In a multivariate analysis, this association remained significant (Table 4).

Patients with the CA genotype had a 2.5 times higher probability for T3 than those with the CC genotype (*p* = 0.032; 95% CI 1.082–5.705). This association remained significant in a multivariate analysis Model No. 2 Table 3 (OR = 2.555 *p* = 0.030; 95% CI 1.094–5.969). Furthermore, carriers of the A allele had a 2.5 times higher probability for T3 than non-carriers (*p* = 0.032; 95% CI 1.082–5.705). In a multivariate analysis, this association remained significant (Table 4).

Lastly, patients with CA genotype had a 12.4 times higher probability for the IIIA stage than those with CC genotype (*p* = 0.001; 95% CI 2.873–53.393). This association remained significant in a multivariate analysis Model No. 2 Table 3 (OR = 12.212 *p* = 0.001; 95% CI 2.786–53.533). The carriers of the A allele had a 12.4 times higher probability for the IIIA stage than non-carriers (*p* = 0.001; 95% CI 2.873–53.393). In a multivariate analysis, this association remained significant (Table 4).

### 3.4. Survival Analysis

**Figure 2:**
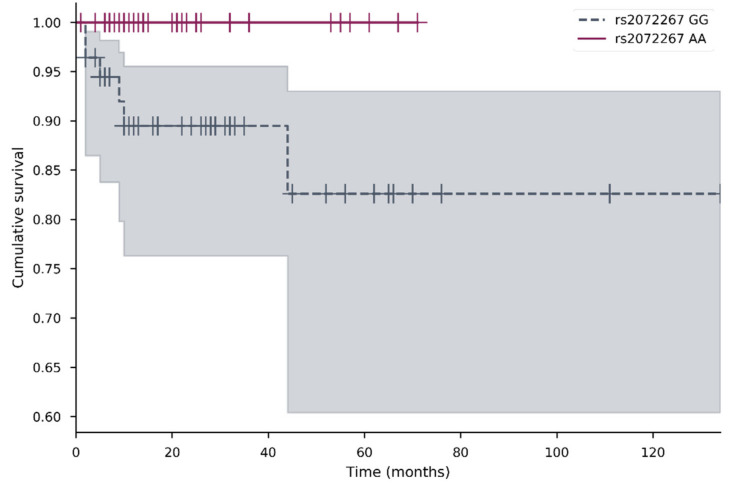
Metastasis-free survival of patients with *POLG* rs2072267 AA and GG genotypes. Kaplan–Meier survival curves for MFS comparing different *POLG* rs2072267 AA and GG genotypes (*p* = 0.032) in the cervical cancer cohort. Censored cases are indicated as a vertical line. The different color represents different genotype; the shadow of the same color represents 95% confidence interval.

The impact of all studied SNVs on survival, including overall survival (OS) and metastasis-free survival (MFS) was analyzed. The OS ranged from 1 to 192 months (median 15); metastasis MFS from 1 to 202 months (average 13). Kaplan–Meier analysis was performed for genotypes and alleles for both OS and MFS. The data suggested that rs2072267 was important for MFS (Figure 2). This was followed by Cox regression analysis; however, no associations were confirmed. No other genotypes or alleles were associated with OS or MFS.

## 4. Discussion

Cervical cancer is the fourth most common cancer in women worldwide and, despite implemented prevention programs, remains prevalent, especially in lower-income countries [1].

It is believed that mitochondria might be important in cancerous processes because they are essential for energy generation and apoptosis in cells [17]. SNVs in nuclear genes, coding for mitochondrial proteins, therefore, might play an important role in mitochondrial functions. These polymorphisms have not been studied enough. We have chosen to analyze the polymorphisms in the nuclear *POLG* gene, which encodes the alpha subunit of polymerase gamma. Polymerase gamma is the only polymerase that is involved in mtDNA replication and repair [11]. To date, only a few studies investigated *POLG* SNVs in breast and colorectal cancer patients [12,13,14]. There is not enough data concerning these SNV effects on patients’ clinical or tumor pathomorphological parameters. In this study, we have focused on association analysis between *POLG* rs3087374, rs2307441, rs2072267, rs976072 SNV, and cervical cancer parameters such as tumor histological type, size, stage, differentiation grade, regional lymph node involvement, distant metastasis, progression, overall and metastasis-free survival.

We decided to analyze SNVs that were not extensively studied before, had low or high allele frequency in the general population, and some that were in the non-coding region of DNA. All of the selected SNVs were predicted to be benign [18,19]. However, in our study, the *POLG* rs3087374 CA genotype was associated with an increased risk of adenocarcinoma histological tumor type, IIIA stage, and T3 tumor size compared to the CC genotype. In the allelic model, A allele of this SNV remained significantly associated with an increased risk of adenocarcinoma. These findings also remained significant in multivariate logistic regression analysis. The rs3087374 C > A is predicted to be a benign, missense (Q1236H) variant [20,21]. The minor allele frequency of this variation in our study was 0.081, similar to the minor allele frequency determined in the 1000 genome project 0.075. The *POLG* rs3087374 was previously investigated in Parkinson’s disease, but no significant associations were determined [21]. In addition, no associations were found between rs3087374 and breast cancer in our previous study [14]. To sum up, the association mentioned above suggests that rs3087374 is linked to cervical cancer pathomorphological parameters such as tumor histological type, stage, size (T), indicating the need for further studies involving different cancer types.

In this study, we found that patients with *POLG* rs2072267 AA genotype had longer metastasis-free survival than those with the GG genotype. This SNV c.2071-22T > C is an intronic *POLG* variant. It is considered to be benign even though it might alter splicing. The G allele frequency of this variation in our study was 0.541, similar to the one in the 1000 genome project 0.465. The *POLG* rs2072267 was also investigated in Parkinson’s disease, ataxia [22,23], colorectal cancer [12], but there were no significant associations determined [21]. Despite this, in our previous study, we found that *POLG* rs2072267 was significantly associated with disease progression in breast cancer patients [14]. The link between *POLG* rs2072267 and disease outcomes from this and our previous study suggests that *POLG* rs2072267 might have a role in cancer progression.

There were also *POLG* rs2307441 and rs976072 single nucleotide variants analyzed in this study, but no significant associations were found. The rs2307441 is a missense variant (c.3428A > G) in the *POLG* coding sequence that leads to Glu to Gly amino acid change (E1143G) at the 1143 protein position. The rs2307441 was studied in Parkinson’s disease [21] and colorectal cancer [12], however, no significant associations were reported. In our previous study, we found it to be associated with tumor vascular invasion and metastasis-free survival in breast cancer patients [14]. Another SNV, rs976072, is in the 3′ end of the *POLG* gene. It was examined in the bladder [24] and breast cancer [14], but no associations were found. However, it was demonstrated to be important for pancreatic cancer [25]. The lack of associations with cervical cancer could indicate that SNVs in POLG rs2307441 and rs976072 are not particularly linked to cervical cancer pathogenesis.

The low sample size and the small number of selected SNVs were the limiting factors of this study. However, we were able to detect a few significant associations which suggest that the *POLG* gene might be important in cervical cancer. We believe that those results could serve as a background for further research on larger and more diverse cancer patient cohorts.

## 5. Conclusions

Our data suggest that mitochondrial polymerase gamma encoded by nuclear *POLG* gene is important for specific tumor phenotype formation and patient outcome in cervical cancer.

## Figures and Tables

**Table 1:** The frequency of *POLG* genotypes and alleles.

*POLG*	N	Genotype	Allele
rs3087374	144	CC—0.837	C—0.919 A—0.081
	28	CA—0.163	
	0	AA—0	
rs2307441	161	TT—0.963	T—0.968 C—0.032
	11	TC—0.064	
	0	CC—0	
rs2072267	42	AA—0.244	A—0.459 G—0.541
	74	AG—0.430	
	56	GG—0.326	
rs976072	58	AA—0.337	A—0.564 G—0.436
	78	AG—0.453	
	36	GG—0.209	

The total count of genotypes (N), together with genotype and allele frequency distribution of *POLG* gene, in the cervical cancer patient group.

**Table 2:** *POLG* gene rs3087374 genotype associations with tumor and clinical data.

Variable	rs3087374		*p*
	CC *n* (%)	CA *n* (%)	
Age Group *			
<56	73 (85.9)	12 (14.1)	0.448
>56	71 (81.6)	16 (18.4)	
T1 tumor size			
No	121 (82.9)	25 (17.1)	0.447
Yes	23 (88.5)	3 (11.5)	
T2 tumor size			
No	71 (80.7)	17 (19.3)	0.269
Yes	73 (86.9)	11 (13.1)	
T3 tumor size			
No	103 (88.0)	14 (12.0)	0.025
Yes	41 (74.5)	14 (25.5)	
T4 tumor size			
No	137 (83.0)	28 (17.0)	0.234
Yes	7 (100)	0 (0.0)	
Cancerous nearby lymph nodes			
No	76 (80.0)	19 (20.0)	0.142
Yes	68 (88.3)	9 (11.7)	
Metastasis			
No	134 (82.7)	28 (17.3)	0.151
Yes	10 (100)	0 (0.0)	
G1 differentiation grade			
No	133 (83.6)	26 (16.4)	0.928
Yes	11 (84.6)	2 (15.4)	
G2 differentiation grade			
No	47 (81.0)	11 (19.0)	0.528
Yes	95 (84.8)	17 (15.2)	
G3 differentiation grade			
No	106 (84.8)	19 (15.2)	0.457
Yes	36 (80.0)	9 (20.0)	
Squamous cell carcinoma			
No	10 (66.7)	5 (33.3)	0.061
Yes	134 (85.4)	23 (14.6)	
Adenocarcinoma			
No	139 (85.3)	24 (14.7)	0.019
Yes	5 (55.6)	4 (44.4)	
Stage IIIA			
No	141 (86.5)	22 (13.5)	0.000
Yes	3 (33.3)	6 (66.7)	
Progress			
No	103 (85.1)	18 (14.9)	0.443
Yes	41 (80.4)	10 (19.6)	
Fact of death			
No	112 (84.8)	20 (15.2)	0.467
Yes	32 (80.0)	8 (20.0)	

* Age groups were assigned by age median. IIIA, stage IIIA of cervical cancer. *n*, numbers. * Age groups were assigned by age median. T, tumor size according to TNM classification; G, tumor differentiation grade.

**Table 3:** Univariate and multivariate logistic regression analysis between SNVs and tumor or clinical data.

Dependent	rs	Covariates	Model No 1				Model No 2			
			Odds	95% CI		*p*	Odds	95% CI		*p*
Adenocarcinoma	rs3087374	CA vs. CC	4.492	1.120	18.012	0.034	4.564	1.050	19.842	0.043
		Age *	1.015	0.963	1.070	0.586	1.015	0.962	1.070	0.586
		T3 (present vs. absent)					0.736	0.138	3.930	0.720
		Stage IIIA (present vs. absent)					1.320	0.096	18.109	0.836
T3	rs3087374	CA vs. CC	2.484	1.082	5.705	0.032	2.555	1.094	5.969	0.030
		Age *	0.984	0.978	0.991	0.000	0.984	0.978	0.991	0.000
		Adenocarcinoma vs. other types					0.775	0.171	3.520	0.742
IIIA	rs3087374	CA vs. CC	12.385	2.873	53.393	0.001	12.212	2.786	53.533	0.001
		Age *	1.022	0.965	1.081	0.459	1.022	0.966	1.082	0.453
		Adenocarcinoma vs. other types					1.164	0.111	12.215	0.899

* Age at the time of diagnosis. T3, size of primary tumor according to TNM classification. IIIA, stage IIIA of cervical cancer. Model No. 1, the logistic regression analysis adjusted for age at diagnosis. Model No. 2, the logistic regression analysis adjusted for age at diagnosis and selected parameters. CI, confidence interval.

**Table 4:** Univariate and multivariate logistic regression analysis between alleles and tumor or clinical data.

Dependent	rs	Covariates	Model No 1				Model No 2			
			Odds	95% CI		*p*	Odds	95% CI		*p*
Adenocarcinoma	rs3087374 A allele	Carrier	4.492	1.120	18.012	0.034	4.564	1.050	19.842	0.043
		Age *	1.015	0.963	1.070	0.586	1.015	0.962	1.070	0.586
		T3 (present vs. absent)					0.736	0.138	3.930	0.720
		IIIA (present vs. absent)					1.320	0.096	18.109	0.836
T3	rs3087374 A allele	Carrier	2.484	1.082	5.705	0.032	2.555	1.094	5.969	0.030
		Age *	0.984	0.978	0.991	0.000	0.984	0.978	0.991	0.000
		Adenocarcinoma vs. other types					0.775	0.171	3.520	0.742
IIIA	rs3087374 A allele	Carrier	12.385	2.873	53.393	0.001	12.212	2.786	53.533	0.001
		Age *	1.022	0.965	1.081	0.459	1.022	0.966	1.082	0.453
		Adenocarcinoma vs. other types					1.164	0.111	12.215	0.899

* Age at the time of diagnosis. T3, size of primary tumor according to TNM classification. IIIA, stage IIIA of cervical cancer. Model No. 1, the logistic regression analysis adjusted for age at diagnosis. Model No. 2, the logistic regression analysis adjusted for age at diagnosis and selected parameters. CI, confidence interval.

## Data Availability

The data presented in this study are available on request from the corresponding author.

## Supplementary Materials

The following are available online at https://www.mdpi.com/article/10.3390/jcm10091838/s1, Table S1: The characterization of cervical cancer patients, Table S2: *POLG* gene rs2307441 genotype associations with tumor and clinical data, Table S3: *POLG* gene rs2072267 genotype associations with tumor and clinical data, Table S4: *POLG* gene rs976072 genotype associations with tumor and clinical data.
