# Supplementary material for: POLG Gene Variants in Cervical Cancer Patients and Their Associations with Clinical and Pathomorphological Tumor Characteristics
Source: J Clin Med. 2021 Apr 23;10(9):1838. doi: 10.3390/jcm10091838 (PMC8123044; doi:10.3390/jcm10091838)
Supplement: Supplementary file 1 [file jcm-10-01838-s001.zip › jcm-1159396-supplementary.pdf]

Table S1. The characterization of cervical cancer patients.

Age		n=172 (100%)
	<56	85 (49.4)
	>56	87 (50.6)
Tumor histological type		
	Squamous cell carcinoma	157 (91.3)
	Adenocarcinoma	9 (5.2)
	Mucinous adenocarcinoma	6 (3.5)
Stage		
	I	16 (9.3)
	II	60 (34.9)
	III	83 (48.3)
	IV	13 (7.5)
Tumor size		
	T1	26 (15.1)
	T2	84 (48.8)
	T3	55 (32)
	T4	7 (4.1)
Grade*		
	G1	13 (7.6)
	G2	112 (65.9)
	G3	45 (26.5)
Metastasis		
	Yes	10 (5.8)
	No	162 (94.2)
Cancerous nearby lymphnodes		
	Yes	77 (44.8)
	No	95 (55.2)
Progress		
	Present	51 (29.7)
	Absent	121 (70.3)
Fact of death		
	Alive	132 (76.7)
	Deceased	40 (23.3)

**Two data points are missing due to unavailable data.*

Table S2. POLG gene rs2307441 genotype associations with tumor and clinical data

Variable	rs2307441		p
	TT<br>n (%)	TC<br>n (%)	
Age group*			
<56	82 (96,5)	3 (3,5)	0,129
>56	79 (90,8)	8 (9,2)	
T1 tumor size			
No	136 (93,2)	10 (6,8)	0,564
Yes	25 (96,2)	1 (3,8)	
T2 tumor size			
No	83 (94,3)	5 (5,7)	0,695
Yes	78 (92,9)	6 (7,1)	
T3 tumor size			
No	110 (94,0)	7 (6,0)	0,747
Yes	51 (92,7)	4 (7,3)	
T4 tumor size			
No	154 (93,3)	11 (6,7)	0,480
Yes	7 (100)	0 (0,0)	
Cancerous nearby lymph nodes			
No	90 (94,7)	5 (5,3)	0,500
Yes	71 (92,2)	6 (7,8)	
Metastasis			
No	152 (93,8)	10 (6,2)	0,631
Yes	9 (90,0)	1 (10,0)	
G1 differentiation grade			
No	148 (93,1)	11 (6,9)	0,327
Yes	13 (100)	0 (0,0)	
G2 differentiation grade			
No	53 (91,4)	5 (8,6)	0,412
Yes	106 (94,6)	6 (5,4)	
G3 differentiation grade			
No	119 (95,2)	6 (4,8)	0,140
Yes	40 (88,9)	5 (11,1)	
Squamous cell carcinoma			
No	15 (100,0)	0 (0,0)	0,289
Yes	146 (93,0)	11 (7,0)	
Adenocarcinoma			
No	152 (93,3)	11 (6,7)	0,421
Yes	9 (100,0)	0 (0,0)	
Progress			
No	113 (93,4)	8 (6.6)	0,858
Yes	48 (94,1)	3 (5,9)	
Fact of death			
No	124 (93,9)	8 (6,1)	0,744
Yes	37 (92,5)	3 (7,5)	

* Age groups were assigned by age median. n – number of cases; T – tumor size according to TNM classification; G – tumor differentiation grade.

Table S3. POLG gene rs2072267 genotype associations with tumor and clinical data

Variable	rs2072267			p
	AA<br>n (%)	AG<br>n (%)	GG<br>n (%)	
Age group*				
<56	23 (27,1)	33 (38,8proc.)	29 (34,1)	0,524
>56	19 (21,8)	41 (47,1)	27 (31,0)	
T1 tumor size				
No	35 (24,0)	64 (43,8)	47 (32,2)	0,875
Yes	7 (26,9)	10 (38,5)	9 (34,6)	
T2 tumor size				
No	20 (22,7)	32 (36,4)	36 (40,9)	0,052
Yes	22 (26,2)	42 (50,0)	20 (23,8)	
T3 tumor size				
No	30 (25,6)	54 (46,2)	33 (28,2)	0,203
Yes	12 (21,8)	20 (36,4)	23 (41,8)	
T4 tumor size				
No	41 (24,8)	72 (43,6)	52 (31,5)	0,365
Yes	1 (14,3)	2 (28,6)	4 (57,1)	
Cancerous nearby lymph nodes				
No	24 (25,3)	38 (40,0)	33 (34,7)	0,663
Yes	18 (23,4)	36 (46,8)	23 (29,9)	
Metastasis				
No	42 (25,9)	70 (43,2)	50 (30,9)	0,079
Yes	0 (0,0)	4 (40,0)	6 (60,0)	
G1 differentiation grade				
No	38 (23,9)	71 (44,7)	50 (31,4)	0,312
Yes	4 (30,8)	3 (23,1)	6 (46,2)	
G2 differentiation grade				
No	10 (17,2)	24 (41,4)	24 (41,4)	0,142
Yes	32 (28,6)	48 (42,9)	32 (28,6)	
G3 differentiation grade				
No	36 (28,8)	51 (40,8)	38 (30,4)	0,111
Yes	6 (13,3)	21 (46,7)	18 (40,0)	
Squamous cell carcinoma				
No	0 (0,0)	8 (53,3)	7 (46,7)	0,066
Yes	42 (26,8)	66 (42,0)	49 (31,2)	
Adenocarcinoma				
No	42 (25,8)	68 (41,7)	53 (32,5)	0,169
Yes	0 (0,0)	6 (66,7)	3 (33,3)	
Progress				
No	32 (26,4)	54 (44,6)	35 (28,9)	0,275
Yes	10 (19,6)	20 (39,2)	21 (41,2)	
Fact of death				
No	36 (27,3)	57 (43,2)	39 (29,5)	0,176
Yes	6 (15,0)	17 (42,5)	17 (42,5)	

* Age groups were assigned by age median. n – number of cases; T – tumor size according to TNM classification; G – tumor differentiation grade.

Table S4. POLG gene rs976072 genotype associations with tumor and clinical data

Variable	rs976072			p
	AA n (%)	AG n (%)	GG n (%)	
Age group*				
<56	31 (36,5)	36 (42,2)	18 (21,2)	0,700
>56	27 (31,0)	42 (48,3)	18 (20,7)	
T1 tumor size				
No	48 (32,9)	68 (46,6)	30 (20,5)	0,744
Yes	10 (38,5)	10 (38,5)	6 (23,1)	
T2 tumor size				
No	30 (34,1)	34 (38,6)	24 (27,3)	0,072
Yes	28 (33,3)	44 (52,4)	12 (14,3)	
T3 tumor size				
No	39 (33,3)	59 (50,4)	19 (16,2)	0,051
Yes	19 (34,5)	19 (34,5)	17 (30,9)	
T4 tumor size				
No	57 (34,5)	73 (44,2)	35 (21,2)	0,356
Yes	1 (14,3)	5 (71,4)	1 (14,3)	
Cancerous nearby lymph nodes				
No	34 (35,8)	39 (41,1)	22 (23,2)	0,441
Yes	24 (31,2)	39 (50,6)	14 (18,2)	
Metastasis				
No	57 (35,2)	71 (43,8)	34 (21,0)	0,202
Yes	1 (10,0)	7 (70,0)	2 (20,0)	
G1 differentiation grade				
No	53 (33,3)	74 (46,5)	32 (20,1)	0,496
Yes	5 (38,5)	4 (30,8)	4 (30,8)	
G2 differentiation grade				
No	15 (25,9)	27 (46,6)	16 (27,6)	0,171
Yes	43 (38,4)	49 (43,8)	20 (17,9)	
G3 differentiation grade				
No	48 (38,4)	53 (42,2)	24 (19,2)	0,137
Yes	10 (22,2)	23(51,1)	12 (26,7)	
Squamous cell carcinoma				
No	2 (13,3)	9 (60,0)	4 (26,7)	0,216
Yes	56 (35,7)	69 (43,9)	32 (20,4)	
Adenocarcinoma				
No	56 (34,4)	74 (45,4)	33 (20,2)	0,585
Yes	2 (22,2)	4 (44,4)	3 (33,3)	
Progress				
No	40 (33,1)	58 (47,9)	23 (19,0)	0,503
Yes	18 (35,3)	20 (39,2)	13 (25,5)	
Fact of death				
No	47 (35,6)	59 (44,7)	26 (19,7)	0,587
Yes	11 (27,5)	19 (47,5)	36 (20,9)	

* Age groups were assigned by age median. n – number of cases; T – tumor size according to TNM classification; G – tumor differentiation grade.
